# Commentary: Crocetin protected human hepatocyte LO2 cell from TGF-β-induced oxygen stress and apoptosis but promoted proliferation and autophagy *via* AMPK/m-TOR pathway

**DOI:** 10.3389/fpubh.2022.1002484

**Published:** 2022-11-14

**Authors:** Ralf Weiskirchen

**Affiliations:** Institute of Molecular Pathobiochemistry, Experimental Gene Therapy and Clinical Chemistry (IFMPEGKC), Rhine-Westphalia Technical University (RWTH) University Hospital Aachen, Aachen, Germany

**Keywords:** crocetin, liver, hepatocyte, cell misidentification, cell culture

In a recent article published in *Frontiers in Public Health*, Guo et al. investigated the therapeutic effects of crocetin on liver injury using the LO2 cell line (1).

Crocetin is a naturally occurring apocarotenoid 20-carbon dicarboxylic acid found in the crocus flower *Crocus sativus L* (Figure 1). The plant also known as saffron is used as a traditional medicine for the treatment of liver disorders (2). The health-promoting effects of crocetin and its derivatives have been confirmed in many other diseases, including hypertension, atherosclerosis, myocardial hypertrophy, arrhythmia, myocardial infarction, myocarditis, coronary artery diseases, stroke, hyperlipidemia, diseases of the nervous system, retinal damage, and many types of cancer (3, 4). This compound can inhibit the growth of cancer cells, most likely through the downregulation of genes involved in inflammation (5). Regarding the liver, it was previously shown that crocetin protects against fulminant experimentally induced hepatic failure and many other hepatic lesions by decreasing apoptosis, inflammation, and oxidative stress in rats and by inhibiting several upstream kinases in cells of hepatic origin (4, 6). In particular, crocetin showed hepatoprotective properties in carbon tetrachloride-induced liver injury in mice *via* induction of antioxidant defense (7). Similarly, crocetin improved Dengue virus-induced liver injury by modulating antioxidant status and reducing the nuclear translocation of NF-κB in Dengue virus-infected mice (8). In a mouse model of non-alcoholic fatty liver disease (NAFLD), crocetin suppressed hepatic fat accumulation and steatohepatitis, and decreased the expression of tumor necrosis factor-α (TNF-α), interleukin-6 (IL-6), and IL-1, while increasing the expression of heme oxygenase 1 (HO-1) and nuclear factor erythroid 2-related factor 2 (Nrf2) that are both key regulators of cellular resistance to oxidants (9). All these studies underpin the hepatoprotective effects of crocetin.

**Figure 1:**
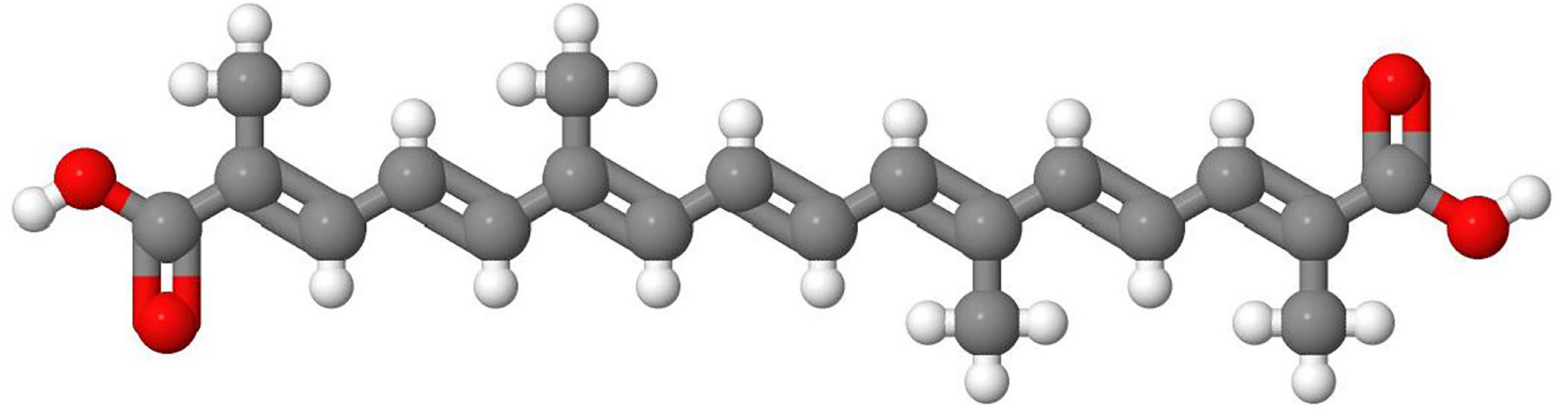
Crocetin, the major active component of saffron. Crocetin (C_20_H_24_O_4_; MW: 328.40 g/mol) is a dicarboxylic acid containing seven conjugated double bonds, 4 side-chain methyl groups, and carboxylic acid groups at both ends. It is a potent antioxidant supposed to be therapeutically effective in many disorders. The chemical structure of transcrocetin (8,8′-Diapocarotenedioic acid, CAS 27876-94-4) depicted here was taken from the PubChem database (CID: 5281232).

Nevertheless, precise mechanistic insights into the molecular activities of crocetin are still missing. As such, the study by Guo et al. aiming to further identify molecular mechanisms by which crocetin evolves its molecular effects is quite reasonable. The authors found that “*Crocetin protected LO2 cells from transforming growth factor-*β*-induced damage by promoting proliferation and autophagy, and suppressing apoptosis and anti-inflammation via regulation of AMPK/m-TOR signaling pathway*” (1). Based on their findings, the authors finally concluded that “*Our results provided clinical prevention and treatment of the occurrence of liver disease with molecular mechanisms*.”

In my view, the study needs critical attention. The authors state that LO2 cells were purchased from the American Type Culture Collection (ATCC) and used as a human hepatocyte cell line. Unfortunately, this assumption is completely wrong. Although the cell line LO2 also known as HL-7702 or L-02 was originally isolated as a human fetal hepatocyte line (10), it was later shown to be a cell line that is cross-contaminated with HeLa cervical cancer cells or even a hybrid of HeLa (11). Like many other cell lines, this false line has been unfortunately used in many studies, thereby provoking tremendous scientific and financial damage, and leading to incorrect or questionable interpretations of “scientific findings.”

Nowadays, this cell line is not anymore available at ATCC, and the Expasy Cellosaurus database lists this cell line under entry CVCL_6926 as a “*Problematic cell line: Contaminated. Shown to be a HeLa derivative*,” while the register of the International Cell Line Authentication Committee (12) (ICLAC-00575) states that “*L-02 was reported to be established from normal liver cells but its STR profile corresponds to HeLa*” (13). Therefore, the presented data solely established with this line have to be taken with caution.

Cell line misidentification is a general serious problem in biomedical research. A conservative estimate found 32,755 articles reporting results with misidentified cells, which in turn were cited by an estimated half a million other papers (14). Another more recent estimate suggested that 8.6% of all cell lines used are problematic cell lines (15). Moreover, it is estimated that 5% of the human cell lines used in manuscripts considered for peer review are misidentified (16). At the same time, the actual ICLAC register released on 8 June 2021 lists nearly 600 cell lines known to be misidentified through cross-contamination or other mechanisms, which once again demonstrates the scale of the problem (12).

Although apparent HeLa cell contamination of human cell lines has already been known for a long time (17) and cell authentication has been widely recommended for many years, the scientific community is still not sensitized enough to recognize the dangers associated with misidentification and cross-contamination (18).

Besides LO2 cells, there are many other misidentified cell lines frequently used in hepatology research (19). Like the L02 cell line, most of them have been shown by short tandem repeat (STR) analysis to be contaminated by or to be identical to HeLa. Prominent hepatic cell lines that are supposed to be misidentified or contaminated with other cell lines include Chang liver cells, GREF-X, HuL-1, WRL 68, and REPC (19). Unfortunately, several scientists still ignore the guidelines and recommendations of the ICLAC to use these lines as derivatives of primary liver cells.

However, the usage of a misidentified cell line in a study does not imperatively mean that the statements or conclusions of the study are questionable. Based on the previously mentioned literature, there is no doubt that crocetin impacts cell viability, cell apoptosis, and cell autophagy by impacting AMPK/m-TOR signaling as supposed by Guo et al. Nevertheless, the overall conclusion is that the results demonstrate that crocetin is a compound that might have therapeutic effects on the pathogenesis of a hepatic disease is not underpinned by any data from this study. Importantly, other studies have very recently shown that crocetin negatively affects bile acid formation and disturbs intestinal homeostasis by promoting inflammation and altering gut microbiota in mice (20), which contradicts the findings of Guo et al.

In sum, the study by Guo et al. would be better titled “*Crocetin protects from TGF-*β*-induced oxygen stress and apoptosis and promotes proliferation and autophagy via the AMPK/m-TOR signaling pathway in LO2/HeLa cells*.” This title would better be suited to summarize the findings of the study and avoid misinterpretation.

## Author contributions

The author confirms being the sole contributor of this work and has approved it for publication.

## Funding

The laboratory of the author was funded by the Germany Research Foundation (grants WE2554/13-1, WE2554/15-1, and WE2554/17-1). The funder had no role in study design, data collection and analysis, decision to publish, or preparation of the manuscript.

## Conflict of interest

The author declares that the research was conducted in the absence of any commercial or financial relationships that could be construed as a potential conflict of interest.

## Publisher's note

All claims expressed in this article are solely those of the authors and do not necessarily represent those of their affiliated organizations, or those of the publisher, the editors and the reviewers. Any product that may be evaluated in this article, or claim that may be made by its manufacturer, is not guaranteed or endorsed by the publisher.
